# Modified structural network backbone in the contralesional hemisphere chronically after stroke in rat brain

**DOI:** 10.1177/0271678X17713901

**Published:** 2017-06-12

**Authors:** Michel RT Sinke, Willem M Otte, Maurits PA van Meer, Annette van der Toorn, Rick M Dijkhuizen

**Affiliations:** 1 Biomedical MR Imaging and Spectroscopy Group, Center for Image Sciences, University Medical Center Utrecht, Utrecht, The Netherlands; 2 Department of Pediatric Neurology, Brain Center Rudolf Magnus, University Medical Center Utrecht, Utrecht, The Netherlands

**Keywords:** Animal models, brain recovery, diffusion tensor imaging, magnetic resonance imaging, stroke

## Abstract

Functional outcome after stroke depends on the local site of ischemic injury and on remote effects within connected networks, frequently extending into the contralesional hemisphere. However, the pattern of large-scale contralesional network remodeling remains largely unresolved. In this study, we applied diffusion-based tractography and graph-based network analysis to measure structural connectivity in the contralesional hemisphere chronically after experimental stroke in rats. We used the minimum spanning tree method, which accounts for variations in network density, for unbiased characterization of network backbones that form the strongest connections in a network. Ultrahigh-resolution diffusion MRI scans of eight post-mortem rat brains collected 70 days after right-sided stroke were compared against scans from 10 control brains. Structural network backbones of the left (contralesional) hemisphere, derived from 42 atlas-based anatomical regions, were found to be relatively stable across stroke and control animals. However, several sensorimotor regions showed increased connection strength after stroke. Sensorimotor function correlated with specific contralesional sensorimotor network backbone measures of global integration and efficiency. Our findings point toward post-stroke adaptive reorganization of the contralesional sensorimotor network with recruitment of distinct sensorimotor regions, possibly through strengthening of connections, which may contribute to functional recovery.

## Introduction

Stroke regularly results in loss of motor function and is one of the main causes of disability in adults worldwide. Nevertheless, most patients experience partial recovery of sensorimotor function in the weeks and months following the stroke, which is believed to be associated with reorganization of surviving neural networks.^1^ This provides opportunities for recovery-promoting therapies that target intrinsic neurorestorative mechanisms.

Post-stroke brain reorganization manifests from micro- (e.g. synaptic plasticity) to macroscale (e.g. cortical remapping). Various studies have revealed local microstructural reorganization around brain lesions after stroke, including dendritic branching, synaptogenesis and neurite outgrowth, which affect networks of neuronal connections at a larger scale.^2–4^ Post-stroke structural remodeling is not limited to perilesional sites and may also extend to the contralesional hemisphere, as has been demonstrated in animal stroke models^5–8^ and human stroke patients.^9–15^ Contralesional structural alterations may be particularly prominent in regions that are homologous to the ipsilesional stroke-affected regions^10,12,15^, and could thereby contribute to partial retrieval of acute stroke-induced loss of specific function.^9–11,14,15^ However, the pattern of remodeling of contralesional neuronal circuitry, particularly at meso- and macroscale, remains largely unresolved.

Post-stroke large-scale neural network alterations can be assessed with neuroimaging techniques, such as magnetic resonance imaging (MRI), which offer powerful means to map structural and functional networks non-invasively in animal models and patients.^1,16^ Today, the gold standard neuroimaging technique to characterize whole-brain structural connectivity in vivo is diffusion-based tractography.^17,18^ Diffusion imaging characterizes the degree and directionality of tissue water diffusion, which informs on the underlying tissue architecture – particularly the arrangement of axonal projections – and allows three-dimensional tracking of neuronal fibers.^18^ A growing amount of data has demonstrated that diffusion-based tractography may inform on alterations in structural brain connectivity after stroke, possibly associated with neuroplasticity.^10,14,15^ However, to our knowledge, so far only one diffusion-based tractography study has reported on contralesional remodeling of network connectivity in stroke patients.^10^

Neural network features are frequently quantified with graph analysis based on nodes (e.g. predefined brain regions) and edges (e.g. connecting fiber tracts).^19–22^ However, graph-based comparison of networks with different edge densities and connectivity distributions remains challenging.^23,24^ Since focal brain injury typically results in less dense structural networks with fewer connected node pairs, alternative network analysis strategies that are not influenced by variations in network size and density could be more appropriate for the characterization of reorganizing networks after stroke. A promising approach is assessment of the network backbone with minimum spanning tree (MST) analysis, which involves a sub-graph of the original network comprising the strongest network connections.^25–27^ Backbone analysis enables unbiased comparison of networks irrespective of differences in density and connectivity distributions, and has been successfully applied to capture network changes during brain development and in brain disorders such as epilepsy, multiple sclerosis, Alzheimer’s disease, brain tumors and schizophrenia.^25,26,28–32^

In the present study, we aimed to identify macroscale alterations in structural network connectivity and organization in the contralesional hemisphere chronically after experimental stroke. To that aim we acquired diffusion MRI data of post-mortem brains from healthy control rats and from rats with sensorimotor deficits after unilateral stroke in the middle cerebral artery territory. Post-mortem imaging allows very long scanning times, enabling acquisition of images with ultrahigh spatial resolution and great structural detail, not feasible under in vivo conditions. We measured white matter volume and integrity in the contralesional hemisphere, and analyzed backbones of diffusion tractography-based structural networks in relation to sensorimotor performance.

## Methods

### Stroke model, sensorimotor function and in vivo MRI

Ethical approval was given by the Animal Experiments Committee of the University Medical Center Utrecht and the Utrecht University, and experiments were performed in accordance with the guidelines of the European Communities Council Directive.

Eighteen adult male Sprague Dawley rats, weighing 280–320 g, were included in the study. Eight rats underwent transient focal cerebral ischemia by 90-min intraluminal occlusion of the right middle cerebral artery, as previously described.^33,34^ Ten rats served as age-matched controls. Behavioral data and in vivo functional MRI data from these animals have been reported previously.^34^

All rats were trained at four and three days before stroke, and behaviorally tested at 2 days before and at 3, 7, 21, 49, and 70 days after stroke. A sensorimotor deficit score was acquired through grading on different subscales of ‘motility, spontaneous activity’ (e.g. moving limbs without proceeding), ‘gait disturbances’ (e.g. walking toward contralateral side), ‘postural signs’ (e.g. forelimb flexion) and ‘limb placing’ (e.g. normal, weak or no placing).^35^ The total sensorimotor deficit score had a range of 0 (i.e. no deficit) to 20 (i.e. maximal deficit) points.

Infarction size and location were assessed at 70 days after stroke on a 4.7T Varian MR scanner with T_2_-weighted MRI (multiple spin echo; repetition time (TR)/echo time (TE) = 3000/17.5 ms; echo train length = 8; field of view = 32 × 32 mm^2^; matrix =128 × 128; 19 coronal slices; slice thickness = 1 mm). Quantitative T_2_ maps were computed as previously described.^34^ Stroke lesions characterized by T_2_ prolongation and tissue liquefaction were manually outlined on T_2_ maps by two independent neuroscientists, and the overlapping area was assigned as infarcted tissue.

### Post mortem anatomical and diffusion MRI

Stroke and control rats were euthanized at day 70 and transcardially perfused with phosphate-buffered saline and 4% paraformaldehyde. After one year of storage in paraformaldehyde, post mortem brains were fixated in a syringe filled with proton-free perfluoropolyether (Fomblin®, Solvay Solexis) to prevent magnetic susceptibility artifacts around the borders of the brain, which facilitates image post-processing.

High angular resolution diffusion imaging of post mortem rat brain was conducted on a 9.4T Varian MR scanner (eight-shot EPI; TR/TE = 6000/32 ms; FOV = 25 × 25 mm^2^; acquisition matrix = 128 × 128; zero-filled to 256 × 256; 91 coronal slices with slice thickness of 0.2 mm; isotropic voxel resolution =0.2 × 0.2 × 0.2 mm^3^; 4 images without diffusion-weighting; diffusion-weighted images in 2 sets of 60 directions equally spaced on a sphere with b = 2871.50 s/mm^2^ in opposite gradient directions (Δ/δ = 13/6 ms); total acquisition time = 13.25 h/dataset).

### Image processing

**Figure 1.**
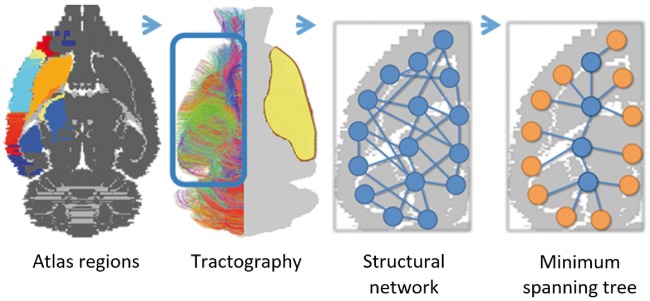
Image processing pipeline. A schematic overview of the image processing steps. After registration with the Paxinos and Watson rat brain atlas, 42 unilateral brain regions were resampled in subject space and used to extract weighted structural networks from diffusion-based tractography in the contralesional hemisphere. A minimum spanning tree (MST) was extracted from the weighted networks and quantified using MST metrics. Atlas labels are plotted on top of a grayscale image of a transverse rat brain slice (first picture). Color-coded contralesional fiber tracts are shown for a single subject (>5 mm tract length only; approximate lesion site depicted in yellow) (second picture). Magnified illustrations display the contralesional cerebral network with nodes and edges (third picture), and corresponding MST (MST leaf nodes in orange) (fourth picture).

Images were pre-processed with FSL 5.0 (http://ww.fmrib.ox.ac.uk/fsl/).^36^ The image processing pipeline as described below is pictured in Figure 1. For each dataset, whole-brain fractional anisotropy (FA) maps were linearly and nonlinearly matched with a custom-built 3D reconstruction of the Paxinos and Watson rat brain atlas,^37^ using affine and nonlinear registrations (FLIRT/FNIRT).^38,39^ FA maps were determined from the diffusion tensor. The contralesional hemisphere was partitioned in 42 cortical and subcortical network regions based on standard anatomical atlas boundaries (see Figure 1) and projected in subject space. Anatomical region details are given in Table 1. A mask that only covered the contralesional hemisphere was created using the R package *oro.nifti* [https://cran.r-project.org/web/packages/oro.nifti/index.html]. This R package provides functions for the input, output and visualization of medical imaging data that follow standard imaging formats. Tractography was done in subject space within the contralesional hemisphere mask.

**Table 1.** Atlas-based network regions.

Atlas description	Paxinos and Watson atlas labels
Agranular insular cortex – dorsal part	AID
Agranular insular cortex – posterior part	AIP
Agranular insular cortex – ventral part	AIV
Amygdaloid nuclei	ACo, BL, BLA, BLP, BLV, BM, BMA, BMP, Ce, CeC, CeL, CeM, CeMAD, CeMAV, CeMPV, IM, La, LaDL, LaVL, LaVM, Me, MeA, MeAD, MeAV, MePD, MePV, PLCo, PMCo
Primary auditory cortex	Au1
Secondary auditory cortex – dorsal area	AuD
Secondary auditory cortex – ventral area	AuV
Cingulate cortex – area 1	Cg1
Cingulate cortex - area 2	Cg2
**Caudate putamen**	**CPu**
Dysgranular insular cortex	DI
Frontal cortex – area 3	Fr3
Granular insular cortex	GI
**Globus pallidus**	**EGP,IGP**
Hippocampus	vhc, dhc, GrDG, CA1, CA2, CA3, Or, MoDG, Py, Rad, SLu, LMol, FC, PoDG
Lateral parietal association cortex	LPtA
**Primary motor cortex**	**M1**
**Secondary motor cortex**	**M2**
Medial parietal association cortex	MPtA
Nucleus accumbens	AcbSh, AcbC, LAcbSh
Piriform cortex	Pir
prelimbic cortex	PrL
Parietal cortex – posterior area – dorsal part	PtPD
Parietal cortex – posterior area – rostral part	PtPR
Retrosplenial cortex	RSGa, RSGb, RSGc, RSD
Rhinal cortex	DIEnt, DLEnt, Ect, Ent, LEnt, MEnt, PRh, VIEnt
Reticular thalamic nucleus	Rt
**Primary somatosensory cortex**	**S1**
**Primary somatosensory cortex – barrel field**	**S1BF**
**Primary somatosensory cortex – dysgranular zone**	**S1DZ**
**Primary somatosensory cortex – oral dysgranular zone**	**S1DZO**
**Primary somatosensory cortex – forelimb region**	**S1FL**
**Primary somatosensory cortex – hindlimb region**	**S1HL**
**Primary somatosensory cortex – jaw region**	**S1J**
**Primary somatosensory cortex – shoulder region**	**S1Sh**
**Primary somatosensory cortex – trunk region**	**S1Tr**
**Primary somatosensory cortex – upper lip region**	**S1ULp**
**Secondary somatosensory cortex**	**S2**
Substantia nigra – reticular part	SNR
Temporal association cortex	TeA
**Thalamus**	**MD, MDC, MDL, MDM, MDPL, PV, PVA, PVP, VPL, VPM, Po, VL**
Visual cortex	V1, V1B, V1M, V2L, V2ML, V2MM

Note: All 42 unilateral rat brain atlas regions (16 sensorimotor network regions in bold) were based on 3D renderings from the Paxinos and Watson rat brain atlas.

### Anatomical characteristics

A white matter mask was constructed using an FA threshold of 0.2 (i.e. all voxels with a subthreshold value were regarded as grey matter). We determined white and grey matter volumes of the contralesional hemisphere. For white matter voxels, we determined the mean FA, radial diffusivity (RD), and axial diffusivity (AD).

### Diffusion tensor-based tractography

Diffusion-based tractography and subsequent connectome reconstructions were performed in MRtrix3® (http://www.mrtrix.org/).^40^ We used diffusion tensor-based tractography to reconstruct whole brain white matter fiber connections on a voxel-wise basis from the diffusion MRI data.^18,41^ Seeds were equally distributed over the whole contralesional hemisphere, from which tracts were generated using a voxel-by-voxel stepwise approach following the principal diffusion directions. Based on our previous work,^42^ we selected tractography parameter settings that yield highest specificity for reconstruction of intrahemispheric connections as suggested for network analyses.^43^ We used an FA threshold of 0.15 (i.e. tract generation was terminated in sub-threshold voxels), a step-size of 130 µm, an angular threshold of 20° (i.e. tracts were terminated when bending with an angle exceeding the threshold) and 25 k streamlines. The relative low FA threshold allowed for optimal tract termination within the cortical gray matter regions.

We used spherical-deconvolution informed filtering of tractograms (SIFT) to improve the accuracy of reconstructed sets of white matter bundles by optimizing and fitting them to the underlying diffusion-weighted images.^44,45^ Thereby, the number of tracts is normalized and corrected for density differences across different regions and datasets.

### Structural network reconstruction

Structural connectivity was determined for each pair of atlas regions. Two regions were considered connected if one or more filtered tracts had their end point in both regions. These were subsequently used to construct a weighted network, represented by the graph *S* = (*N*, *W*) where *N* is the set of 42 unilateral network regions and *W* = *w_ij_* is the *N* × *N* weight matrix, where *w_ij_* is set to the SIFT-corrected number of all tracts connecting the region pairs. Self-connections were excluded. The filtered number of tracts was used as a measure of connectivity strength. The density of undirected networks, with no self-connections, was defined as the ratio of the number of existing edges over the total possible number of edges (that is, *N* × (*N –* 1)/2)).

Weighted connectivity matrices were formed for 42 × 42 atlas regions. To examine inter-subject consistency of connections, group-based average structural networks were generated from the prevalence of connections (after binarization of individual network matrices (i.e. all connection weights were set to a value of one)) within the control and stroke group.

### Sensorimotor network reconstruction

We extracted 15 sensorimotor regions from the acquired structural network matrices, i.e. the primary motor cortex (M1), secondary motor cortex (M2), caudate putamen, thalamus, globus pallidus, sub-regions of the primary somatosensory cortex (S1) (i.e. forelimb region, oral dysgranular zone, upper lip region, shoulder region, hindlimb region, dysgranular zone, trunk region, barrel field, jaw region and S1 not otherwise specified) and secondary somatosensory cortex (S2). Sensorimotor connectivity matrices were formed from 16 × 16 unilateral sensorimotor regions.

### MST analysis

For all rats’ graphs *G*, the MST was calculated using the Prim–Jarník algorithm. Every graph *G* has a set of nodes *N*, an edges set *W* and a real valued cost $\ell {\;}_{w}$ assigned to each edge w∈W, i.e. the connectivity cost is the inversed edge weight along the connection. A spanning tree, defined as subset of the network nodes forming graph *G* which connects all nodes and does not contain any cycles or loops,^27^ was computed for all unilateral (contralesional) structural network reconstructions. A MST *T* minimizes the sum of the costs of its edges, $\ell (T)=\sum_{w\in T}\ell_{w}$ over the set of all possible MSTs on *G*.^46^ Group-based MST connectivity matrices were generated from the prevalence of MST connections (after binarization of individual MST network matrices) for the control and stroke groups.

The following metrics were calculated for the MSTs at nodal or network level:

- *Betweenness centrality (nodal):* an often used network measure of hubness,^21^ which is based on the number of shortest paths passing through a node. Betweenness centrality of a node increases with the number of those passages and is defined as ${\mathrm{bc}}_{i}=\frac{1}{\left(n-1)(n-2\right)}\sum_{j\neq k,k\neq i,j\neq i}n\frac{g_{\mathrm{jk}}(i)}{g_{\mathrm{jk}}}$ where $g_{\mathrm{jk}}$ is the shortest path between two nodes of the MST and $g_{\mathrm{jk}}(i)$ is the number of node paths that pass actually through node *i*.
- *Strength (nodal)*: the tree node strength is a summation of all nodal connection weights.^21,47^
- *Leaf number (N_leaf_) (network):* the number of tree nodes having exactly one connection to another tree node. A higher leaf number reflects increased global network integration and efficiency.^25,26^
- *Diameter (d) (network):* the largest distance between any two nodes of the tree, ranging from 2 to *M* = *N* – 1, where *M* is the number of connections in the tree. The largest possible diameter decreases with increasing leaf number *N_leaf_* (i.e. improved network integration or global efficiency).^25,26^
- *Eccentricity (network):* the shortest path length between a tree node *I* and any other node from the tree. Eccentricity decreases when nodes become more central in the tree.^25,26^
- *Kappa (network):* width of degree distribution: *Κ* = < *k*^2 ^> / < *k* > .^25^ This measure reflects the vulnerability to hub-node damage. Networks characterized with a scale-free degree distribution,^48^ e.g. neural networks, have particularly high Kappa values.

### Contralesional network backbone metrics versus lesion size and sensorimotor outcome

We related network backbone characteristics to lesion size and sensorimotor deficit score at day 70, using univariable linear regression. A *p-*value < 0.05 was considered statistically significant.

### Software

Network analyses, statistical modelling and generation of some figures were performed in *R 3.2* (http://www.r-project.org/) using the packages *igraph*, *reshape2, network, lattice, oro.nifit, plyr, sna, lm* and *ggplot2*.

## Results

### Post mortem anatomical and diffusion MRI data

**Figure 2.**
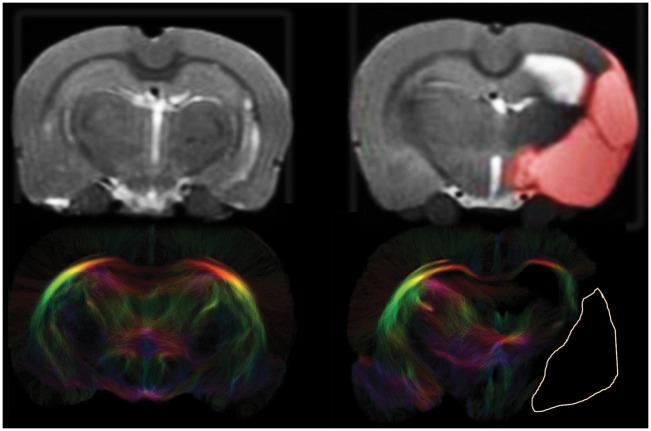
Anatomical images and tractograms of control and stroke rat brain. Coronal views of in vivo anatomical T_2_ maps (top) and corresponding post mortem diffusion-based tractograms (bottom) of a control and post-stroke rat brain slice (T_2_-weighted and diffusion MRI sequences are described in the Methods section). The unilateral ischemic lesion area is depicted by a red overlay in the anatomical image and delineated by a white line in the tractogram. Loss of fiber connections is clearly visible in the ipsilesional hemisphere of stroke animals. In the contralesional hemisphere, fiber density was comparable to that in controls.

Post-mortem MRI 10 weeks after stroke (N = 8) displayed ischemic lesions in the subcortical and cortical MCA territory (Figure 2, top row). Mean lesion volume was 264 ± 72 mm^3^. Individual lesion volumes are shown in Table S1.

We found no significant change in volumes of the contralesional white matter, gray matter or total hemisphere of stroke animals as compared to controls (Table 2). Furthermore contralesional white matter FA, RD and AD values were not significantly different from control values (Table 2).

**Table 2.** Gray and white matter characteristics for control and stroke animals.

Anatomical characteristic	Controls (N = 10)	Stroke (N = 8)
Total volume (mm^3^)	909 ± 47	890 ± 40
Gray matter volume (mm^3^)	373 ± 98	326 ± 70
White matter volume (mm^3^)	536 ± 60	564 ± 38
Fractional anisotropy	0.34 ± 0.01	0.34 ± 0.01
Radial diffusivity (×10^−3^ mm^2^/s)	0.21 ± 0.03	0.22 ± 0.02
Axial diffusivity (×10^−3^ mm^2^/s)	0.35 ± 0.05	0.37 ± 0.04

Figure 2 (bottom row) shows representative examples of tractography-based structural connectome reconstructions of control and post-stroke rat brain. Loss of fiber connections is clearly visible in the ipsilesional hemisphere where the lesion area is characterized by complete absence of fibers. In the contralesional hemisphere of stroke animals, fiber density was slightly (1.1 ± 0.01 %) higher than in control animals (*t* = 2.05, *p* = 0.06).

### Group-based structural network and MST connectivity matrices

**Figure 3.**
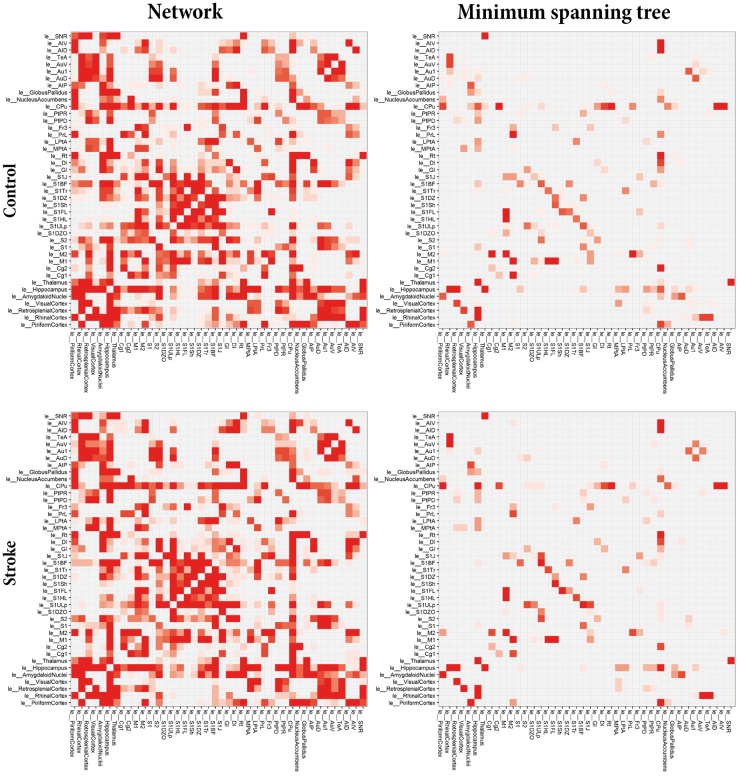
Average connectivity matrices for the total networks and minimum spanning trees. Average structural connectivity matrices of total networks (left) and MST backbones (right) of the left (contralesional) hemisphere for control (top) and stroke (bottom) animals. Connectivity weights are based on prevalence of connections for each group, ranging from low (white) to high (red). ‘le_[Name]’ indicates node in left (contra-lesional) hemisphere. The MSTs, reflecting the backbone connections, primarily consisted of connections between sensorimotor regions, such as the primary motor cortex, the secondary motor cortex, caudate putamen and the forelimb and hindlimb regions of the primary somatosensory cortex. Overall, the connectivity pattern in the contralesional hemisphere of stroke animals was quite similar to its counterpart in control animals, although some subtle differences are apparent in the connectivity matrices.

Figure 3 shows group-based average connectivity matrices for the total structural network and the network backbones (MST) for the left (contralesional) hemisphere of stroke and control animals (see Figure S1 for representative examples of individual weighted networks and corresponding backbones). Some structural connections are highly stable (high prevalence), whereas existence of other connections varies across individual animals (low prevalence). The MSTs, reflecting the backbone connections, primarily consisted of connections between sensorimotor regions, such as the primary motor cortex, the secondary motor cortex, caudate putamen and the forelimb and hindlimb regions of the primary somatosensory cortex. Overall, the connectivity pattern in the contralesional hemisphere of stroke animals was quite similar to its counterpart in control animals. However, subtle differences were apparent in the connectivity matrices. For example in the caudate putamen, which revealed connections with the ventral area of the secondary auditory cortex and the primary auditory cortex in the stroke group but not in the control group. Also, for the backbone connections, we observed connections (e.g. between the upper lip region and jaw region of the primary somatosensory cortex or between the secondary motor cortex and piriform cortex) in the stroke group that were not apparent in controls. However, no significant differences in group-based average network backbone characteristics were found between the left, contralesional hemisphere in stroke animals and the left hemisphere in control animals (Figure S2).

### Nodal MST metrics

**Figure 4.**
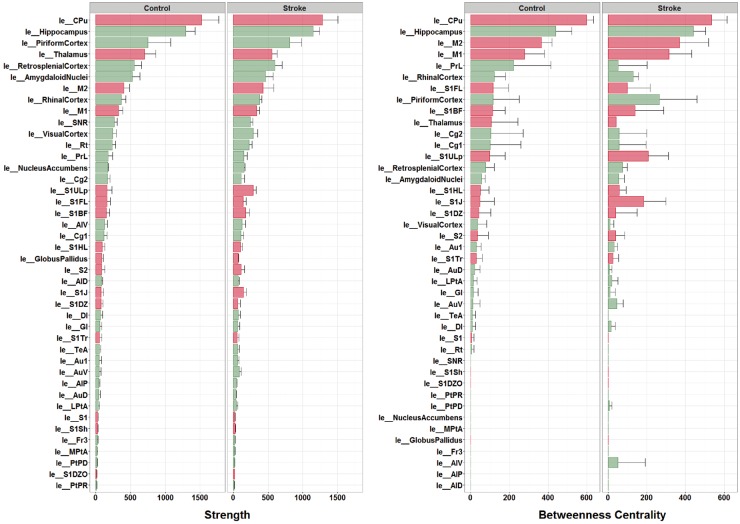
Node strengths and betweenness centralities from individual networks. Node strength (left) and betweenness centrality (right) (mean ± standard deviation) – calculated from MSTs of the left (contralesional) hemisphere in individual control and stroke animals – ranked from high to low (based on control group data). ‘le_[Name]’ indicates brain atlas region in left (contralesional) hemisphere. Red bars represent sensorimotor regions. The caudate putamen and hippocampus were the strongest connected nodes in control as well as stroke animals. The upper lip and jaw region of the primary somatosensory cortex displayed considerable modifications, i.e. increased node strength in stroke animals. The caudate putamen, the hippocampus, the primary motor cortex and the secondary motor cortex were the four most significant hub-nodes, for control as well as stroke animals. Some regions displayed increased (e.g. the upper lip and jaw regions of the primary somatosensory cortex) or decreased betweenness centrality (e.g. the pre-limbic cortex) after stroke.

Figure 4 (left) shows the ranking of individual node strengths measured from MSTs from the individual control and stroke animals. The caudate putamen and hippocampus were the strongest connected nodes in control as well as stroke animals. The upper lip and jaw region of the primary somatosensory cortex displayed considerable modifications, i.e. increased node strength (Figure 4) and degree (Figure S3), in stroke animals.

Ranking of individual node betweenness centralities (i.e. hubness) (Figure 4, right) showed that the caudate putamen, the hippocampus, the primary motor cortex and the secondary motor cortex were the four most significant hub-nodes, for control as well as stroke animals. Nevertheless, some regions displayed increased (e.g. the upper lip and jaw regions of the primary somatosensory cortex) or decreased betweenness centrality (e.g. the pre-limbic cortex) after stroke.

### Network backbone metrics versus lesion size and functional outcome

**Figure 5.**
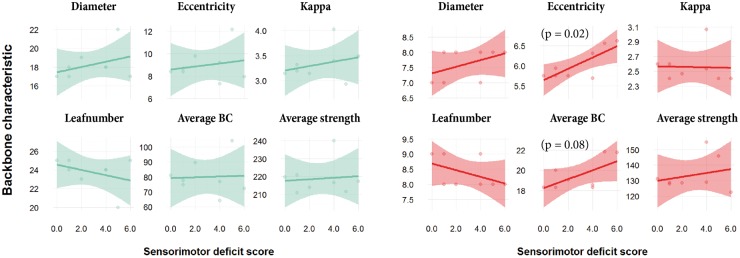
Contralesional backbone metrics versus sensorimotor deficit score. Linear model fits of MST metrics for the total structural network (left, green) and the sensorimotor network (right, red) in the contralesional hemisphere versus sensorimotor deficit score at day 70 after stroke (transparent bands indicate standard deviation). BC: betweenness centrality. Sensorimotor deficit score was not significantly correlated with MST backbone characteristics of the total structural network. However, sensorimotor deficit score was positively associated with eccentricity and average betweenness centrality of the specific MST from the contralesional sensorimotor network.

Linear regression revealed no trends or significant association between contralesional network backbone metrics at post-stroke day 70 and lesion volume (Figure S4). Also, sensorimotor deficit score at day 70 (shown in Table S1) was not significantly correlated with MST backbone characteristics of the total structural network (Figure 5, left). However, sensorimotor deficit score was positively associated with eccentricity (*p* = 0.02) and average betweenness centrality (*p* = 0.08) of the specific MST from the contralesional sensorimotor network (Figure 5, right).

## Discussion

To characterize the configuration of structural network backbones in the contralesional hemisphere chronically after stroke, we analyzed high-resolution post-mortem MRI data of rat brains collected at 70 days after unilateral stroke. Structural backbones, assessed with an unbiased network analysis approach based on MSTs, were relatively consistent across control and stroke animals, but modifications in nodal importance of specific sensorimotor network regions were apparent in post-stroke brains. Furthermore, we found that sensorimotor outcome was associated with backbone metrics related to network efficiency.

Changes in the organization of neural networks in the contralesional hemisphere after stroke may reflect remote tissue degeneration as well as remote tissue plasticity in response to focal ischemic injury.^1–4,10,16,49^ In our study, we measured an increase in connectivity strength and hubness of certain contralesional sensorimotor regions (e.g. upper lip and jaw regions of the primary somatosensory cortex) in brains from rats that were sacrificed at a plateau stage of sensorimotor recovery after stroke. These findings suggest adaptive reorganization of the contralesional sensorimotor network with recruitment of distinct sensorimotor regions, possibly through strengthening or formation of connections. This corresponds with our previous findings of enhanced in vivo functional connectivity (part of which measured in the same group of stroke animals^34^) and stronger neuroanatomical connectivity between contralesional sensory and motor regions.^34,50^ In the current study, the observed increased consistency, strength and hubness of network backbone regions in stroke animals may particularly denote strengthening of existing connections. In an earlier diffusion MRI study in which network communicability was measured chronic stroke patients, several regions with increased communicability were identified in the contralesional hemisphere.^10^ Yet, contralesional regions with decreased communicability were also observed, which may be related to secondary degeneration of transhemispheric white matter pathways.^14^ However, diffusion MRI data from our study in rats did not reveal significant changes in white matter volume or integrity in the contralesional hemisphere.

Sensorimotor outcome at day 70 was negatively associated with eccentricity and average betweenness centrality of specifically the sensorimotor part of the contralesional network backbone (no significant correlation was found for the backbone of total contralesional network). This implies that low integration and global efficiency (i.e. high network eccentricity), and strong reliance on highly connected nodes (i.e. high average betweenness centrality) in the contralesional sensorimotor network may impede recovery of sensorimotor function after stroke, which would further emphasize the importance of efficient (modified) network functioning in the contralesional hemisphere of recovering stroke patients.

Despite the potential of network backbone analysis for reliable and robust assessment of the strongest and most important connections, we cannot draw conclusions about the contribution of other structural connections that may have played a role in network remodeling in our study. Furthermore, although our post mortem dataset offered high-quality reconstruction of structural connections, it was limited to a single chronic time-point after stroke, which did not allow us to look into earlier structural network changes when functional recovery is particularly ongoing, i.e. in the first weeks after stroke. Nevertheless, our study shows that modifications in structural network backbones at meso- to macroscale level can be identified from high-resolution diffusion-based tractography in rat brain. We measured alterations in MST metrics in the contralesional hemisphere chronically after stroke, which we could relate to the degree of functional outcome.

The relative voxel size as compared to total rat brain volume (8 × 10^−3^ mm^3^/900 mm^3^) in our post mortem study, is comparable to that of in vivo DTI studies in humans (8–27 mm^3^/1500 × 10^3^ mm^3^). It would be valuable, for translational purposes, to assess structural network backbone configurations with DTI in stroke patients. Furthermore, longitudinal studies could help to resolve the temporal pattern of structural network alterations in relation to loss and recovery of functions. Alternative network methods, based on generative models, may be employed to further elucidate structural repair mechanisms in post-stroke brain at whole-connectome level. These include Bayesian exponential random graph models,^51,52^ or mixed-effect models^53^ and Gibbs distribution models^54^, which also allow comparison of networks with different densities. Insights from these studies may aid in the development and selection of new treatment strategies for patients recovering from stroke.

## Supplementary Material

Supplementary material
