# Supplementary material for: Modified structural network backbone in the contralesional hemisphere chronically after stroke in rat brain
Source: J Cereb Blood Flow Metab. 2017 Jun 12;38(9):1642–53. doi: 10.1177/0271678X17713901 (PMC6120129; doi:10.1177/0271678X17713901)
Supplement: Supplementary material [file JCB713901_supplementary_material.pdf]

Supplementary Figures

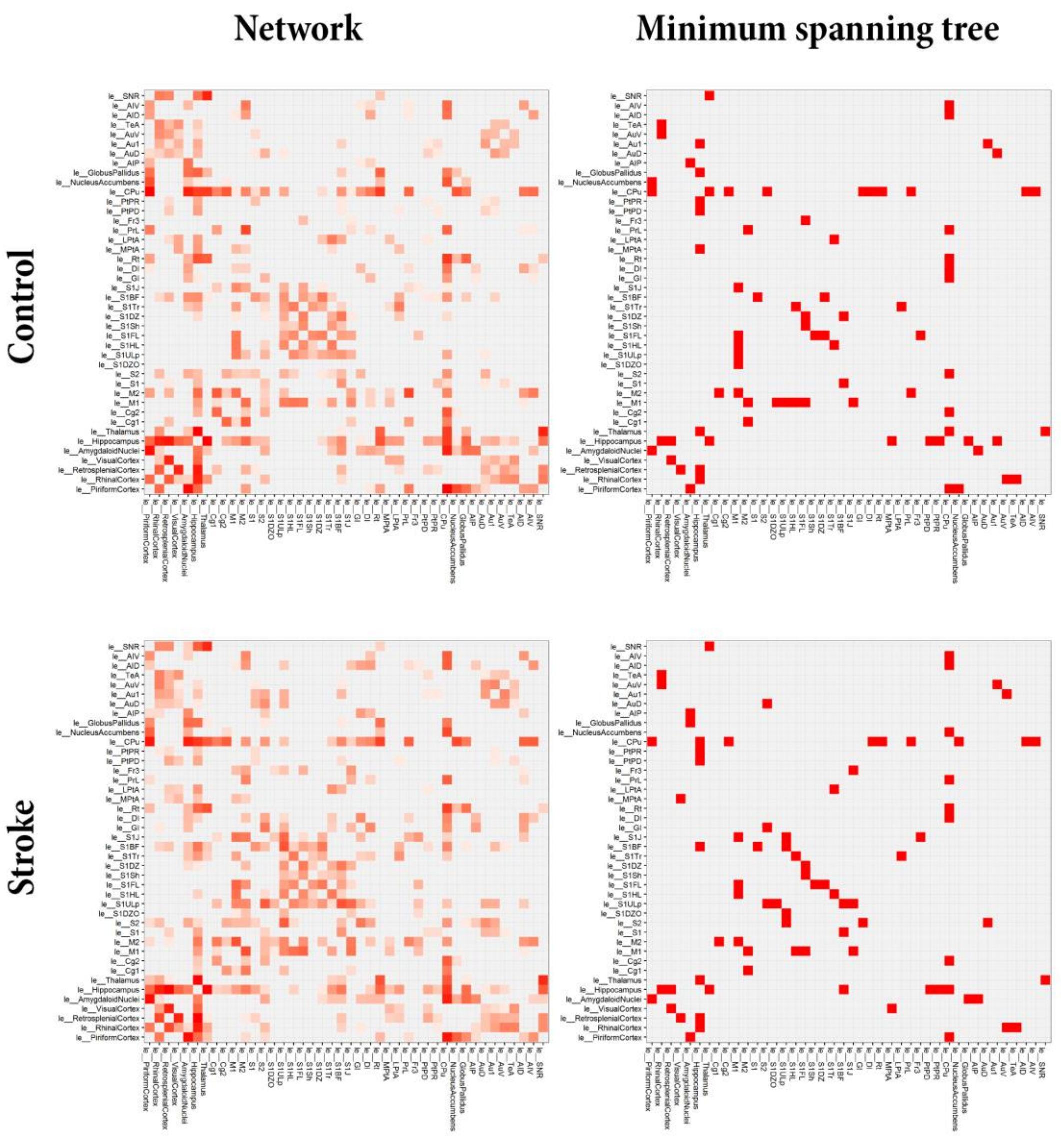

Figure S1. Individual weighted connectivity matrices for the total network and minimum spanning tree. Representative weighted connectivity matrices of total networks (left) and MST backbones (right) of the left (contralesional) hemisphere of a control (top) and a stroke (bottom) animal. Connectivity weights are based on number of tractography streamlines between regions, ranging from low (white) to high (red). ‘le_[Name]’ indicates node in left (contralesional) hemisphere.

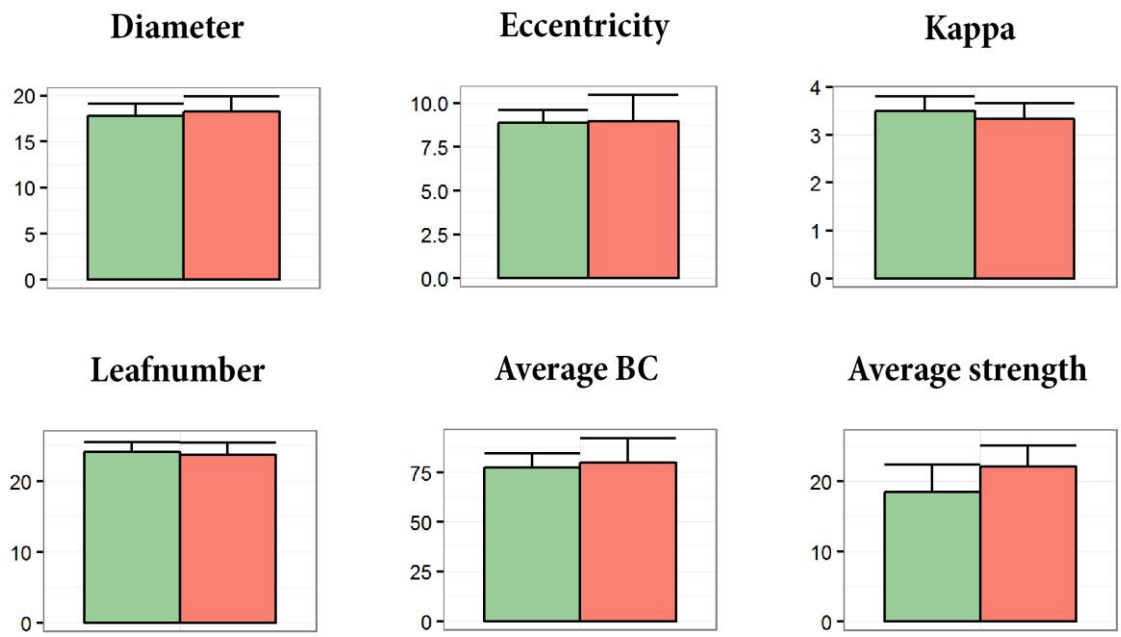

Figure S2. Network backbone characteristics. Network backbone metrics (mean ± standard deviation) calculated from group-based average MSTs of the left (contralesional) hemisphere in the control (green) and stroke (red) animals. BC: betweenness centrality.

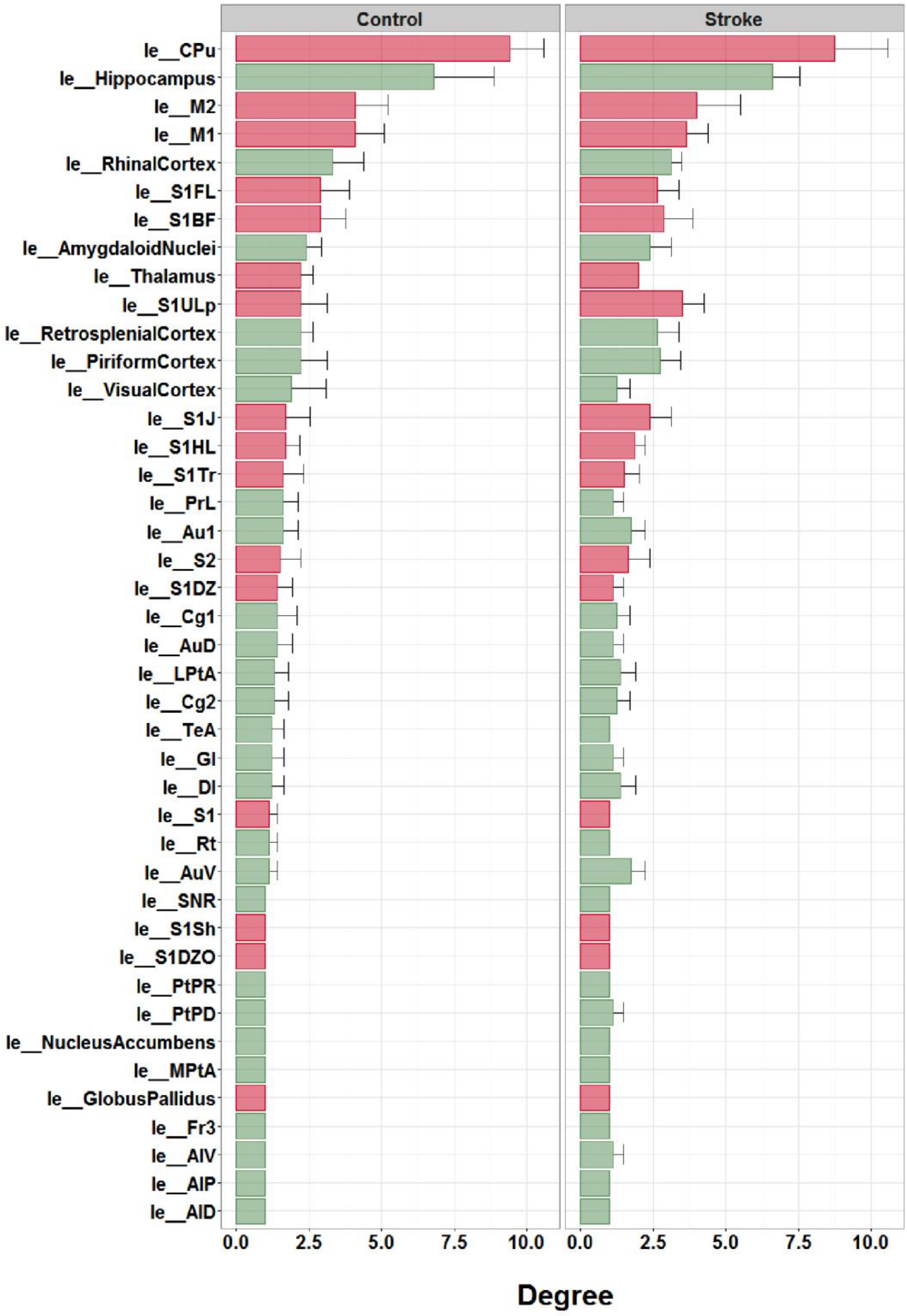

Figure S3. Node degrees from backbone networks. Betweenness centralities (left) and node strength (right) (mean ± standard deviation) –calculated from MSTs of the left (contralesional) hemisphere in control and stroke animals– ranked from high to low (based on control group data). 'le_[Name]' indicates brain atlas region in left (contralesional) hemisphere. Red bars represent sensorimotor regions.

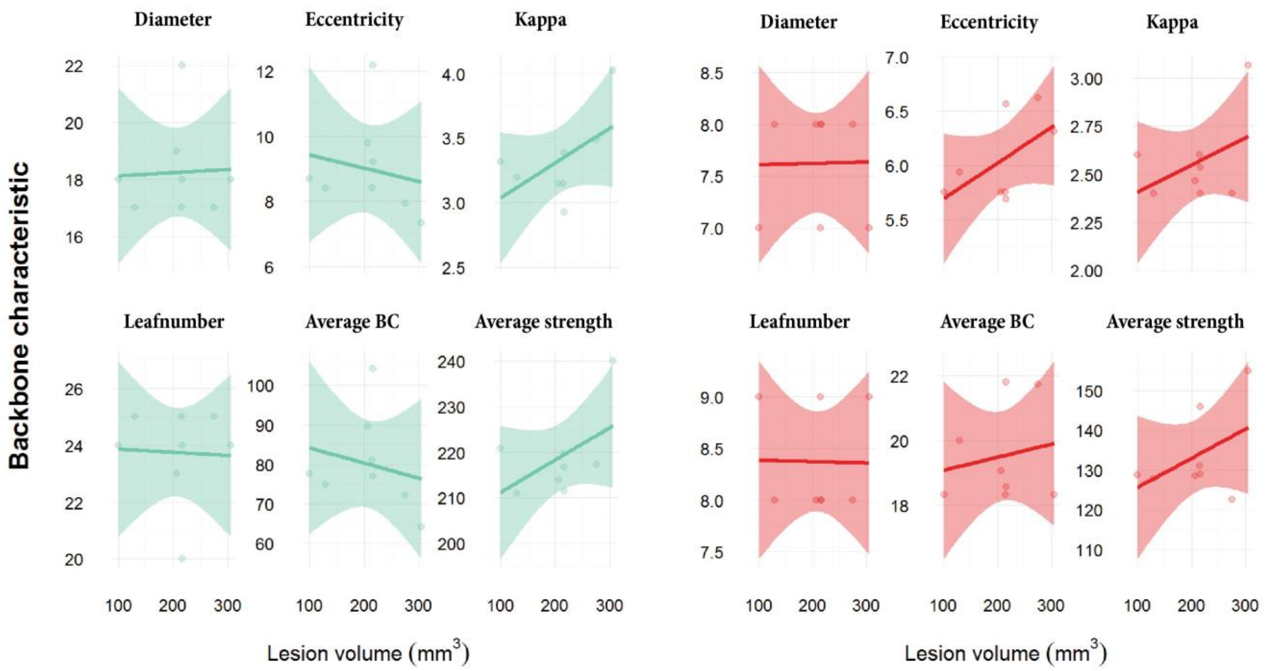

Figure S4. Contralesional MST metrics versus lesion volume. Linear model fits of MST metrics versus lesion volume for the total structural network (left, green) and the sensorimotor network (right, red) in the contralesional hemisphere (transparent bands indicate standard deviation). BC: betweenness centrality.

Supplementary Tables

Table S1. Features of individual rats at 70 days after stroke.

Rat	Ischemic damage	Lesion volume (mm^3)	Sensorimotor deficit score
1	Subcortical + cortical	206	2
2	Subcortical + cortical	275	6
3	Subcortical + cortical	100	1
4	Subcortical + cortical	216	5
5	Subcortical + cortical	214	0
6	Subcortical + cortical	304	4
7	Subcortical + cortical	216	4
8	Subcortical + cortical	129	1
